# Effects of substance use disorder on oxidative and antioxidative stress markers: A systematic review and meta‐analysis

**DOI:** 10.1111/adb.13254

**Published:** 2022-11-23

**Authors:** Thiago Wendt Viola, Rodrigo Orso, Luisa Fossati Florian, Miguel Gomes Garcia, Marco Giovanni Signor Gomes, Eduarda Mascarenhas Mardini, João Paulo Ottolia Niederauer, Aline Zaparte, Rodrigo Grassi‐Oliveira

**Affiliations:** ^1^ Developmental Cognitive Neuroscience Lab, School of Medicine, Brain Institute of the Rio Grande do Sul (BraIns) Pontifical Catholic University of Rio Grande do Sul (PUCRS) Porto Alegre Brazil; ^2^ LSU Pulmonary, Critical Care & Immunology, Department of Medicine Louisiana State University of Health Sciences New Orleans Louisiana USA; ^3^ Translational Neuropsychiatry Unit, Department of Clinical Medicine Aarhus University Aarhus Denmark

**Keywords:** aging, antioxidants, illicit drugs, meta‐analysis, oxidative stress, substance‐related disorders

## Abstract

Recently, it has been suggested that central and peripheral toxicities identified in persons with substance use disorder (SUD) could be partially associated with an imbalance in reactive oxygen species and antioxidant defenses. We conducted a systematic review and meta‐analysis to investigate whether SUD is associated with oxidative stress and to identify biomarkers possibly more affected by this condition. We have included studies that analysed oxidant and antioxidant markers in individuals with SUD caused by stimulants, alcohol, nicotine, opioids, and others (cannabis, inhalants, and polysubstance use). Our analysis showed that persons with SUD show higher oxidant markers and lower antioxidant markers than healthy controls. SUD was associated specifically with higher levels of oxidant markers malondialdehyde, thiobarbituric acid reactive substances and lipid peroxidation. Conversely, the antioxidant superoxide dismutase and the total antioxidant capacity/status were lowered in the SUD group. A meta‐regression analysis revealed that persons with alcohol use disorder had higher oxidative stress estimates than those with stimulant use disorder. Moreover, individuals evaluated during abstinence showed smaller antioxidant effect sizes than non‐abstinent ones. Our findings suggest a clear oxidative imbalance in persons with SUD, which could lead to cell damage and result in multiple associated comorbidities, particularly accelerated aging.

## Graphical abstract

This meta‐analysis found that substance use disorders are related to increased oxidant and reduced antioxidant markers, suggesting a clear oxidative imbalance. Meta‐regressions showed that alcohol use disorder is related to higher oxidative stress estimates in comparison with stimulant use disorder and that individuals on abstinence showed smaller antioxidant effect sizes in comparison with those with current use.

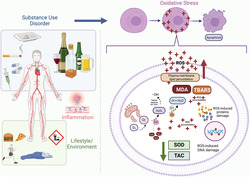

## 1 INTRODUCTION

Substance use disorder (SUD) is a cluster of cognitive, behavioural and physiological symptoms elicited by continued substance use despite significant associated problems.^1^ Several drugs may be involved in the development of SUD, such as alcohol, cannabis, hallucinogens, inhalants, opioids, sedatives, hypnotics, anxiolytics, stimulants (amphetamine‐type substances, cocaine, etc.), and tobacco. Alcohol use accounts for 2.8 million annual deaths worldwide, whereas misuse of illicit drugs accounts for 450,000 deaths.^2^ Because chronic relapses characterize SUD, the life course of patients often includes multiple attempts at treatment,^3^ risky health behaviours^4^ and years of exposure to stress and adverse experiences.^5^ Issues related to polysubstance use and comorbid neuropsychiatric conditions are also widespread.^6^ In people with SUD, the combination of psychosocial factors and the pharmacological properties of drugs could potentiate brain and nervous system toxicity and cellular damage.^7^

Stress hormones, inflammation and oxidation are the main physiological mechanisms involved in SUD‐induced multi‐systemic toxicity.^7^ Reactive oxygen species (ROS) and reactive nitrogen species (RNS) are unstable, highly reactive molecules formed during oxygen metabolism.^8^ They play a role in enzymatic reactions, mitochondrial electron transport, signal transduction, activation of nuclear transcription factors, gene expression and the antimicrobial action of neutrophils and macrophages. However, the reducing environment inside healthy cells is maintained by antioxidant enzymes regulating and preventing free radical‐mediated damage.^8^ This reducing environment is supported by the action of antioxidant enzymes and substances, such as superoxide dismutase (SOD), catalase (CAT), glutathione peroxidase (GSH‐Px) and others.^9^ Alterations in the redox state and depletion of antioxidants due to high exposure to oxygen‐ or nitrogen‐reactive species lead to oxidative stress (OS) and oxidative injury.^10^ The use of cannabis, alcohol, amphetamines, cocaine, opiates and tobacco may cause OS, contributing to SUD‐induced toxicity in the brain and peripheral nervous system.^11^ Furthermore, a higher proportion of oxidant markers compared with antioxidants in SUD patients at different stages of treatment might lead to cell dysfunction and death through oxidation of DNA, proteins or phospholipids,^10^ with important clinical implications for premature morbidity and mortality.

Previous studies identified a wide diversity of oxidant/antioxidant markers altered by SUD, with mixed findings highlighted in qualitative literature reviews.^7^, ^10^, ^11^, ^12^ A quantitative analysis is essential to provide a clearer picture of the relationship between SUD and OS. Thus, we conducted a meta‐analysis to test whether SUD is associated with OS and to identify which biomarkers are more affected by SUD and might be used as an early indicator of SUD‐induced OS responses. We also used meta‐regression techniques to explore the heterogeneity of published findings, testing the effects of SUD clinical features and methodological factors in OS estimates.

## 2 METHODS

### 2.1 Search methodology

We retrieved the relevant studies on 4 December 2020, from three online databases: PubMed, Web of Science and Scopus. We used the following terms related to oxidant/antioxidant markers and SUDs: [‘redox’ OR ‘oxidative stress’ OR ‘antioxidant’ OR ‘anti‐oxidant’ OR ‘antioxidant enzyme’ OR ‘anti‐oxidant enzyme’ OR ‘total antioxidant capacity’ OR ‘total anti‐oxidant capacity’ OR ‘total antioxidant potential’ OR ‘anti‐oxidant capacity’ OR ‘superoxide dismutase’ OR ‘glutathione peroxidase’ OR ‘catalase’ OR ‘paraoxonase’ OR ‘glutathione reductase’ OR ‘malondialdehyde’ OR ‘8‐F2‐isoprostane’ OR ‘protein carbonyl’ OR ‘nitrosative stress’ OR ‘nitrative stress’ OR ‘nitro‐oxidative stress’ OR ‘nitrooxidative stress’ OR ‘nitro oxidative stress’ OR ‘thiobarbituric acid reactive substances’ OR SOD OR CAT OR ‘glutathione disulfide’ OR ‘glutathione’ OR ‘thiobarbituric reactive substances’ OR ‘reactive nitrogen species’ OR ‘reactive oxygen species’] AND [‘Drug dependence’ OR ‘Addiction’ OR ‘Substance‐related disorders’ OR ‘substance use disorder’ OR ‘substance use addiction’ OR ‘substance use dependence’ OR ‘drug abuse’ OR ‘substance abuse’ OR ‘addiction’ OR ‘drug abuse’ OR ‘alcohol use disorder’ OR ‘cocaine use disorder’ OR ‘opioid use disorder’ OR ‘tobacco use disorder’ OR ‘cannabis use disorder’ OR ‘amphetamine use disorders’ OR ‘Inhalant use disorder’ OR ‘polysubstance dependence’]. The study followed the Cochrane recommendations for developing a search strategy.^13^

### 2.2 Screening and eligibility

**FIGURE 1:**
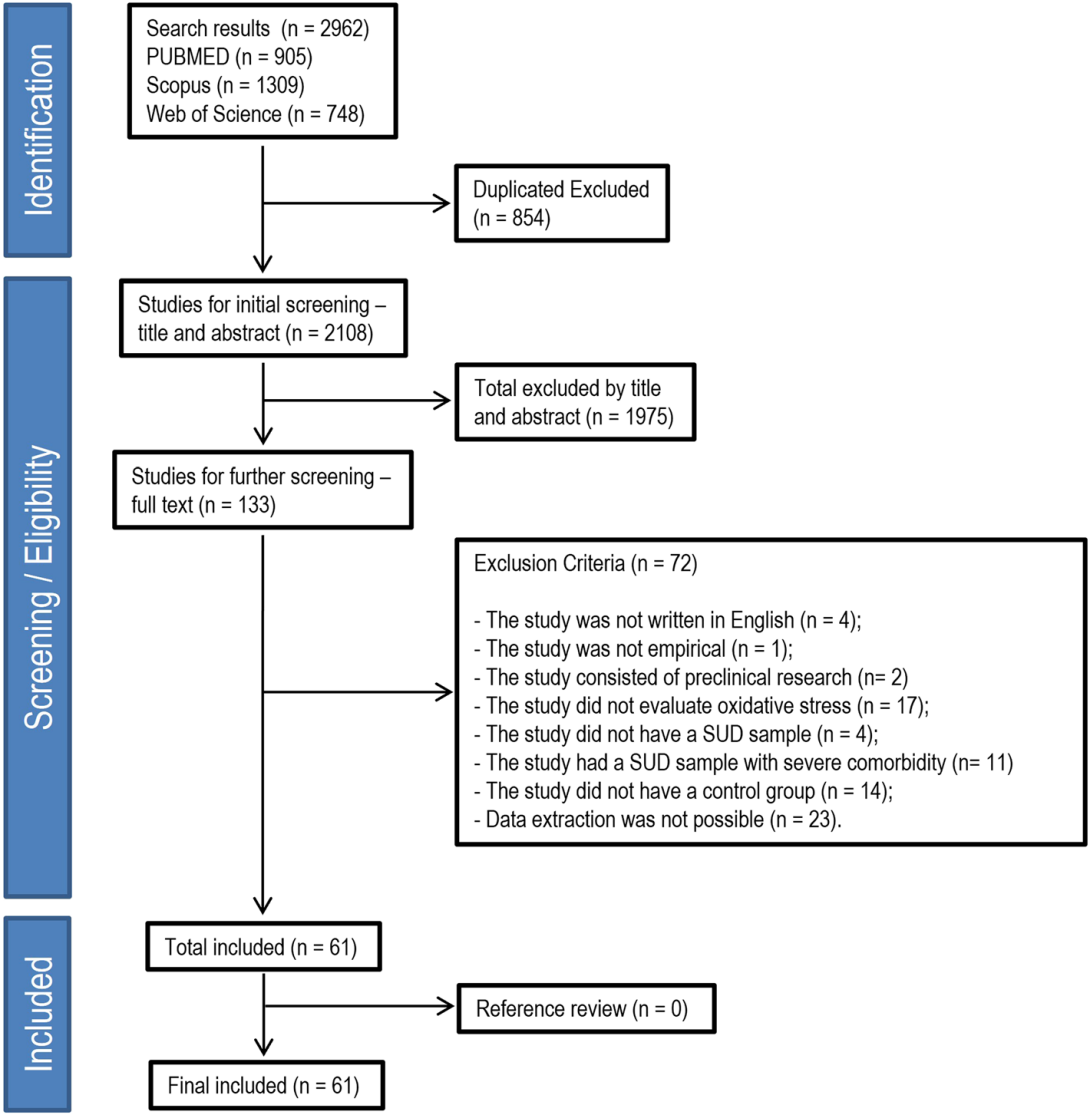
Flowchart of the systematic review. SUD, substance use disorder

All references were imported to Rayyan (https://rayyan.qcri.org), a free web application for the management of systematic reviews. The articles were then selected in three steps. The first step was to exclude duplicate studies using the Rayyan software. The second step was to screen the titles and abstracts for studies designed around a quantitative framework for data analysis (e.g., longitudinal studies, case‐control studies and cross‐sectional studies). The studies based on animal models were excluded from this selection. In the third step, full‐text articles were assessed for eligibility based on the following exclusion criteria: (1) The study was not written in English; (2) the study was not empirical; (3) the study was designed as a preclinical research; (4) the study did not evaluate OS markers; (5) the study did not specifically sample SUD cases (e.g., subjects who used/tried substances of abuse but did not have an SUD); (6) the study used SUD samples with a severe associated medical comorbidity (e.g., HIV/AIDS); (7) absence of a healthy control group; and (8) studies from which data extraction was not possible. All selection steps were executed independently by four authors (EM, LF, MS and MG). In the first two steps, articles were screened using the Rayyan Software, whereas in the last step, each article was individually assessed. Any disagreements on the inclusion or exclusion of studies during the process were resolved based on a consensus between the senior authors (RO and TWV). Figure 1 shows the flowchart of this systematic review.

### 2.3 Data extraction

The following data were extracted from all included studies by four independent authors (EM, LF, MS and MG): ‘first author’, ‘publication year’, ‘study design’, ‘sample size’, ‘percentage of females’, ‘mean age’, ‘drug of preference’, ‘diagnostic instrument’, ‘lifetime substance use’, ‘oxidative/antioxidant stress marker’, ‘analysis method’, ‘biological sample’, and ‘assessment period’. Some studies included the percentage of individuals who reported regular use of different substances, even if they had a drug of preference, so we also recorded this data. We extracted the mean and standard deviations for oxidant/antioxidant markers in healthy controls and SUD groups. If other values (median, standard error or interquartile range) had been reported instead, the mean and standard deviations were estimated as follows: (1) median as mean; (2) standard error multiplied by the square root of sample size, as standard deviation; and (3) interquartile range divided by 1.35, as standard deviation. Data available only in graph form instead of text were extracted using the Getdata software (version 2.26.0.20, © S.Fedorov).

Importantly, the clinical diagnoses of SUD across studies were based on the following methods: clinical interviews based on criteria of the Diagnostic and Statistical Manual of Mental Disorders (DSM), urine tests for drug screening associated with self‐reported data and clinical screening instruments such as the Addiction Severity Index (ASI) the Alcohol Use Disorders Identification Test (AUDIT), the Fargeström Test for Nicotine Dependence, the Cut, Annoyed, Guilty and Eye Questionnaire (CAGE), the Short Michigan Alcohol Screening Test and self‐reported data only. Similar methods were used for the identification of healthy controls.

### 2.4 Coding of potential moderators

The following variables and codes were used as potential moderators for the meta‐analysis:

- Drug of preference, coded as (0) stimulant (cocaine, methamphetamine and amphetamine); (1) alcohol; (2) nicotine; (3) opioid (heroin and opium); and (4) others (less frequently studied drugs included in the review, such as cannabis, inhalants and polysubstance use without a preferred substance);
- Biological sample, coded as (0) serum; (1) brain tissue; (2) plasma; and (3) others (e.g., erythrocytes, bronchoalveolar lavage fluid and cerebrospinal fluid);
- Lifetime substance use, coded as (0) less than 5 years and (1) more than 5 years of usage (according with evidence showing that illicit drug use will be associated with more physical health comorbidities after 5 years of regular consumption^14^);
- Assessment period, coded as (0) abstinence (including detoxification treatment); (1) non‐abstinence and (2) post‐mortem;
- Age of SUD samples: continuous variable;
- Percentage of females in SUD sample: continuous variable;
- Age differences between control and SUD samples, coded as (0) less than 5 years of mean age differences between groups and (1) more than 5 years of mean age differences between groups.
- Female percentage differences between control and SUD samples, coded as (0) less than 10% of differences between groups and (1) more than 10% of differences between groups.

### 2.5 Data analysis

The estimated effect size of oxidant and antioxidant stress markers was determined using a standardized mean difference (SMD) formula (Cohen's *d*). First, meta‐analytical models were generated for oxidant and antioxidant markers as a general grouped analysis. We then generated meta‐analytical models for specific markers (e.g., thiol levels [TLs], SOD and nitric oxide [NO]). All meta‐analyses were conducted using a multilevel approach based on the restricted maximum likelihood estimator (REML) method. The REML assumes a multivariate normal distribution for the random‐effects model. With a two‐level hierarchical data structure, samples within studies were nested within samples between studies. We chose the multilevel approach because some studies could contribute with more than one sample, which violated the assumption of independence between outcomes. This strategy allowed us to account for the heterogeneity between studies and for the fact that some of them reported data from independent SUD subgroups, that is, comparing more than one SUD subgroup to the same control group, resulting in multiple effect sizes. Following the Cochrane guidelines, we therefore counteracted an overweighting of the effect sizes by dividing the sample size of the shared control group by the number of comparisons with independent clinical groups from the same study.

Q‐statistics were used to test for heterogeneity between studies. Sources of heterogeneity were explored via subgroup analyses and meta‐regression models with the inclusion of potential moderators. These were carried out for the grouped meta‐analysis (oxidant and antioxidant) with a large number of studies and as many effect sizes as possible. Therefore, we assured that subgroup analyses included at least 10 effect sizes by level/group of potential moderators, as per the recommendations of Spineli and Pandis.^15^ The estimated proportional reduction in the total variance attributed to potential moderators of heterogeneity was computed using the variance accounted for (VAF), a pseudo R‐squared value.

Publication bias was detected using funnel plots' asymmetry and further statistically proven by Egger's regression test. Outlier and influential case diagnostics were carried out using the externally standardized residuals, difference in fits (DFFITS) values, Cook's distances, covariance ratios, leave‐one‐out estimates of the amount of heterogeneity, leave‐one‐out heterogeneity test statistics, hat values and weights. All statistical analyses were performed using the ‘metafor’ package (version 2.4‐0), an open‐source add‐on for the statistical software environment R (version 4.0.0).

## 3 RESULTS

The initial search yielded 2962 potentially relevant studies. After excluding duplicate records (*n* = 854), we screened the remaining 2108 studies. Title and abstract screenings resulted in 133 studies for a full‐text review. After reading the full‐text articles, we excluded 74 studies based on the exclusion criteria. In the final screening stage, 61 studies were included in this review, and detailed methodological information about each study is presented in Supporting information. Figure 1 shows the flowchart of this systematic review. The datasets used and analysed during the current study are available from the corresponding author upon reasonable request.

### 3.1 Characteristics of studies

The combined sample size of the included studies was 10 373 participants (5167 from healthy control [HC] groups and 5206 from SUD groups). The mean number of participants in the healthy group was 81.25 (SD ± 236.31), ranging from 3 to 1886. The mean number of participants in the SUD group was 62.72 (SD ± 74.48), ranging from 5 to 335. The mean age in the healthy control group was 34.45 years (SD ± 9.1), and 41.42 years (SD ± 8.05) in the SUD group. The average percentage of females in the healthy control group was 17.72%, whereas in the SUD group, it was 14.46%. Interestingly, the publication year of studies ranged from 1992 to 2020, showing that the effects of SUD on OS markers have been a topic of research since the early 1990s.

The drug of preference was divided into five groups: stimulants (18 studies), opioids (15 studies), alcohol (12 studies), nicotine (10 studies) and other substances (10 studies). While cannabis has been extensively studied across multiple fields, our review identified very few studies on cannabis in the context of OS; we, therefore, merged it with other substances. The clinical diagnoses of SUD across studies were based on the following methods: clinical interviews based on criteria of the DSM (25 studies), urine tests for drug screening associated with self‐reported data (10 studies), clinical screening instruments such as the Addiction Severity Index, AUDIT, Fargestrom, CAGE and the Short Michigan Alcohol Screening Test (eight studies) and self‐reported data only (nine studies). Nine studies were carried out as post‐mortem investigations.

Biological samples were collected from the following materials: serum (26 studies), plasma (12 studies), brain tissue (four studies), others (seven studies; lung, alveolar macrophages, cerebrospinal fluid, erythrocytes, red blood cells and hemolysate) or more than one biological material (12 studies). The samples were collected from abstinent participants (14 studies) and non‐abstinent ones (32 studies), whereas 15 studies assessed OS in both abstinence stages, and five were carried out post‐mortem. It is worth noting that 77% of the studies that evaluated patients in abstinence from their drug of preference combined it with abstinence from alcohol.

Table 1 shows all the data (definition, number of studies and effect sizes) on oxidant and antioxidant markers as analysed in the included studies. Biomarkers were grouped into 13 categories: TL; SOD; NO; lipid peroxidation (LP); total antioxidant capacity/status (TACS); malondialdehyde (MDA); protein oxidation (PO); thiobarbituric acid reactive substances (TBARSs); CAT; reduced glutathione (GSH); GSH‐Px; glutathione reductase (GR); and gamma‐glutamyl transferase (GGT).

**TABLE 1:** Biomarker definition

Included biomarkers	Grouped marker	Definition	Oxidant/antioxidant	*N*	*K*
Total thiol levels	Thiol levels (TLs)	Thiols are those compounds that contain the sulphhydryl group (−SH) attached to a carbon atom. Thiols are efficient antioxidants biomarkers and protect cells from damage by free radicals. Both intracellular and extracellular redox states of thiols play an essential role in protein structure and function, regulation of enzymatic activity of transcription factors and antioxidant protection.	Antioxidant	4	9
Thiol disulphide homeostasis					
Protein thiol content					
Native thiol					
Superoxide dismutase	Superoxide dismutase (SOD)	SODs are a group of metalloenzymes and form the first line of defence against reactive oxygen species damage. They are proteins that catalyse the dismutation of superoxide anion free radical (O_2_^−^) into molecular oxygen and hydrogen peroxide (H_2_O_2_) and decrease O_2_^−^ levels in cells.	Antioxidant	25	45
*SOD1*					
*SOD2*					
Copper–zinc superoxide dismutase					
Nitric oxide	Nitric oxide (NO)	NO is a neurotransmitter. It is a small, labile, lipid‐permeable free radical molecule. NO plays an important role in the normal function and the dysfunction of the brain, showing cytoprotective and cytotoxic properties.	Antioxidant	7	8
Nitric oxide metabolites					
Nitric oxide synthase					
Isoprostane	Lipid peroxidation (LP)	Lipid peroxidation can be described as a process in which oxidants attack lipids that contain carbon–carbon double bonds, especially polyunsaturated fatty acids (PUFAs). Also, the oxidative stress caused by ROS production or free radicals can inflict direct damage to lipids, leading to three step reaction: initiation, propagation and termination.	Oxidant	8	11
8‐Isoprostane					
8‐Iso‐prostaglandin F2alpha					
Lipid peroxidation					
Lipid oxidation					
4‐Hydroxynonenal					
Cyclooxygenase‐2					
Trolox equivalent antioxidant capacity	Total antioxidant capacity/status (TACS)	TAC and TAS are types of measurements for antioxidants in the body. TAC is the measure of the amount of free radicals scavenged by a test solution, and it is used to evaluate the antioxidant capacity of biological samples. Likewise, TAS is used to measure the overall antioxidant status of the body.	Antioxidant	13	14
Total reactive antioxidant potential					
Total antioxidant scavenging activity					
Total antioxidant capacity					
Total antioxidant status					
Malondialdehyde‐acetaldehyde	Malondialdehyde (MDA)	MDA is generated by the peroxidation of PUFAs. MDA is also manufactured in the process of prostaglandin synthesis. Therefore, MDA is the product of oxidative stress that leads to lipid peroxidation, causing cell walls to shatter and lipid membranes to oxidize into MDA.	Oxidant	24	40
Malondialdehyde					
Protein carbonyl contents	Protein oxidation (PO)	Protein oxidation is the process that occurs modifications of a protein induced either by ROS or indirect reactions by oxidative stress secondary products. Oxidative reactions that affect proteins can change their physical and chemical properties, such as conformation, structure, solubility, susceptibility to proteolysis and enzyme activities.	Oxidant	10	14
Advanced oxidation protein product					
Carbonyl					
Thiobarbituric acid reactive substances	Thiobarbituric acid reactive substances (TBARSs)	TBARSs are aldehydes generated by the ROS‐induced degradation of PUFAs. These aldehydes are a subproduct of lipid peroxidation. Also, TBARS is an assay that detects lipid peroxidation and measures MDA.	Oxidant	10	16
Catalase	Catalase (CAT)	CAT is one of the crucial antioxidant enzymes that fights against oxidative stress by destroying cellular hydrogen peroxide to produce water and oxygen and maintaining an optimum level of the molecule in the cell, essential for cellular signalling processes. CAT deficiency can play an important role in the pathogenesis of several human diseases (e.g., Parkinson's disease, diabetes mellitus, schizophrenia and bipolar disorder).	Antioxidant	15	19
Glutathione	Reduced glutathione (GSH)	Glutathione is an antioxidant that protects cells from ROS. GSH is responsible for reducing any disulphide bond formed within cytoplasmic proteins to cysteines. GSH is often used with oxidized glutathione to measure the ratio of cellular toxicity.	Antioxidant	12	26
Reduced glutathione					
Glutathione peroxidase	Glutathione peroxidase (GSH‐Px)	GSH‐Px is a cytosolic enzyme with the capacity to scavenge free radicals. It also catalyses the reduction of hydrogen peroxide to water and oxygen and the reduction of peroxide radicals to alcohol and oxygen. Furthermore, increasing the endogenous levels of GSH‐Px could also have a crucial role to resolve oxidative‐stress‐induced pathologies.	Antioxidant	12	25
Glutathione reductase	Glutathione reductase (GR)	GR is a flavin adenine dinucleotide (FAD)‐dependent protein. GR can maintain glutathione in the reduced form by restoring intracellular GSH, which is a consequence of reduced GSSG in the presence of NADPH and FAD.	Antioxidant	3	9
NADPH quinone oxidoreductase					
Gamma‐glutamyl transferase	Gamma‐glutamyl transferase (GGT)	GGT has multiple functions including catalytic transfer of y‐glutamyl groups to amino acids and shorts peptides and hydrolysis of GSH to gamma‐glutamyl fraction and cysteinylgylcine in GSH and GSH conjugate catabolism. It is an enzyme that is expressed in many organs, such as liver, kidney, pancreas and bile ducts, and can be affected by adverse drug reactions.	Oxidant	4	14
C‐Glutamyltranspeptidase					

*Note*: Definition of oxidant and antioxidant biomarkers analysed.

Abbreviation: NADPH, reduced nicotinamide adenine dinucleotide phosphate; ROS, reactive oxygen species.

### 3.2 Grouped meta‐analysis of SUD effects on oxidant and antioxidant markers

**FIGURE 2:**
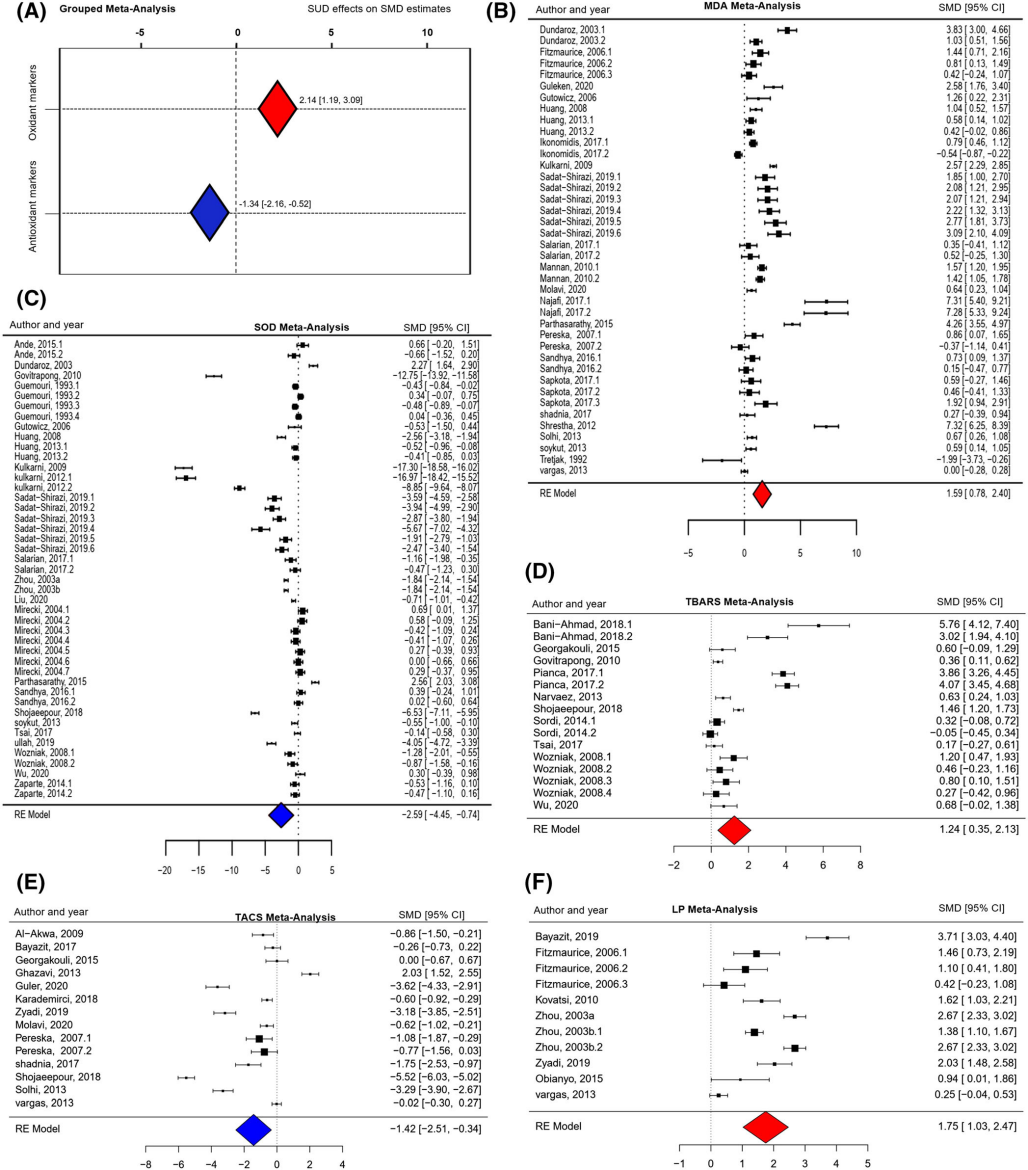
Forest plots. A: Grouped meta‐analysis of substance use disorder (SUD) effects on oxidant/antioxidant markers; B: malondialdehyde (MDA) meta‐analysis; C: superoxide dismutase (SOD) meta‐analysis; D: thiobarbituric acid reactive substance (TBARS) meta‐analysis; E: total antioxidant capacity status (TACS) meta‐analysis; F: lipid peroxidation (LP) meta‐analysis. Negative values indicate a significant reduction whereas positive values indicate a significant increase; red indicates oxidant markers; blue indicates antioxidant markers. CI, confidence interval; RE, random‐effects; SMD, standardized mean difference

In the first analysis, the full dataset was split into oxidant and antioxidant stress markers according to Table 1 specifications. These grouped meta‐analyses revealed increased oxidant levels (estimate = 2.14; SE = 0.48; 95% CI = 1.19/3.09; *p* ≤ 0.0001) and decreased antioxidant defences (estimate = −1.34; SE = 0.41; CI = −2.16/−0.52; *p* = 0.0013) due to SUD, as shown in Figure 2A. Our analyses revealed significant heterogeneity for both meta‐analyses (Q‐test *p*‐value ≤ 0.0001). Additionally, funnel plots showed that studies were not symmetrically distributed (Supporting information), suggesting that asymmetry could be attributed to publication bias, methodological differences or true heterogeneity.^16^ The Egger's test was significant (*p* = 0.0001) for both meta‐analyses.

Outlier/influential case detection methods were then applied, showing three significant effect sizes for oxidant meta‐analyses and five for antioxidant analyses. The recalculated results without outliers were still significant (oxidant estimate = 1.14; SE = 0.15; CI = 0.84/1.45; *p* ≤ 0.0001; antioxidant estimate = −0.90; SE = 0.24; CI = −1.38/−0.42; *p* = 0.0002), confirming the reliability of the conclusions of the grouped meta‐analysis. However, this procedure allowed us to assume that our initial results had been overestimated: when removing outliers, the mean values of oxidant levels caused by SUD might have increased by 1.14 SD units instead of 2.14 SD. Similarly, the mean values of antioxidant defence levels might have decreased by 0.90 SD instead of 1.34 SD. The sources of heterogeneity in the grouped meta‐analysis were further explored in Section 3.4.

### 3.3 Meta‐analysis of SUD effects on specific oxidant and antioxidant stress markers

The meta‐analysis for specific OS markers revealed an increase in levels of MDA (estimate = 1.58; SE = 0.41; CI = 0.78/2.39; *p* = 0.0001) (Figure 2B), TBARS (estimate = 1.23; SE = 0.45; CI = 0.35/2.12; *p* = 0.0062) (Figure 2D) and LP (estimate = 1.74; SE = 0.36; CI = 1.02/2.46; *p* ≤ 0.0001) (Figure 2F) due to SUD. No significant differences were found for GGT and PO (all *p*‐values >0.05).

The meta‐analysis for specific antioxidant markers revealed a decrease in SOD (estimate = −2.59; SE = 0.94; CI = −4.44/−0.73 *p* = 0.0062) (Figure 2C) and TACS (estimate = −1.42; SE = 0.55; CI = −2.50/−0.34; *p* = 0.0099) (Figure 2E) levels due to SUD. No significant differences were found for CAT, GR, GSH, GSH‐Px, NO and TL (all *p*‐values >0.05).

### 3.4 Subgroup analysis and meta‐regressions

#### 3.4.1 Drug of preference

All subgroup analysis and meta‐regression data are presented in Table 2. We compared studies and their derived effect sizes according to the drug of preference reported by SUD samples. Stimulants were used as a reference category due to its frequent presence across studies. We found that the drug of preference was not a significant moderator of heterogeneity for grouped meta‐analyses of oxidant and antioxidant stress markers. However, for oxidant markers, there was a significant difference between stimulants and alcohol, suggesting that alcohol was associated with increased effect sizes and larger SMDs.

**TABLE 2:** Subgroup analysis and meta‐regression models

	Coefficient	SE	95% CI	*p*‐Value	VAF (%)
Oxidant markers					
Drug of preference				0.1861	6.36%
Stimulant (*k* = 31)	Reference				
Alcohol (*k* = 19)	2.8468	1.2720	0.35 to 5.33	**0.0252**	
Nicotine (*k* = 14)	0.2235	1.4776	−2.67 to 3.11	0.8797	
Opioid (*k* = 18)	0.8989	1.2390	−1.52 to 3.32	0.4681	
Other (*k* = 13)	0.2067	1.3718	−2.48 to 2.89	0.8802	
Percentage of alcohol users				**0.0341**	8.76%
Less than 100% (*k* = 48)	Reference				
100% of the sample (*k* = 20)	2.5600	1.2909	0.08 to 5.03	**0.0423**	
Biological sample				0.1475	4.35%
Serum (*k* = 35)	Reference				
Brain tissue (*k* = 21)	−2.2626	1.4677	−5.13 to 0.61	0.1232	
Plasma (*k* = 28)	−2.2878	1.0875	−4.41 to −0.15	**0.0354**	
Other (*k* = 11)	−1.8859	1.3357	−4.50 to 0.73	0.1580	
Lifetime substance use				0.8846	1.87%
Less than 5 years (*k* = 44)	Reference				
More than 5 years (*k* = 20)	0.0731	0.5031	−0.91 to 1.05	0.8846	
Assessment period				0.8846	0.01%
Abstinence (*k* = 26)	Reference				
Non‐abstinence (*k* = 48)	0.4672	0.7851	−1.07 to 2.01	0.5518	
Post‐mortem (*k* = 21)	−0.8699	1.5426	−3.89 to 2.15	0.5728	
Age of SUD sample (*k* = 89)	0.0008	0.0488	−0.09 to 0.09	0.9862	0.01%
Age differences between HC and SUD				0.5597	0.01%
Less than 5 years (*k* = 84)	Reference				
More than 5 years (*k* = 5)	−1.3580	2.3282	−5.92 to 3.20	0.5597	
Percentage of females in SUD sample (*k* = 90)	−0.0233	0.0169	−0.05 to 0.01	0.1695	4.55%
Female % differences between HC and SUD				0.4629	0.85%
Less than 10% (*k* = 73)	Reference				
More than 10% (*k* = 17)	−1.0854	1.4784	−3.98 to 1.81	0.4629	
Antioxidant markers					
Drug of preference				0.4373	6.94%
Stimulant (*k* = 65)	Reference				
Alcohol (*k* = 37)	−1.7534	1.1621	−4.03 to 0.52	0.1313	
Nicotine (*k* = 13)	0.5725	1.2778	−1.19 to 3.07	0.6541	
Opioid (*k* = 28)	−0.3187	1.0953	−2.46 to 1.82	0.7710	
Other (*k* = 12)	−1.0290	1.4951	−3.95 to 1.90	0.4913	
Biological sample				0.5902	3.73%
Serum (*k* = 41)	Reference				
Brain tissue (*k* = 40)	0.3201	1.5276	−2.67 to 3.31	0.8340	
Plasma (*k* = 33)	0.8101	1.0220	−1.19 to 2.81	0.4280	
Other (*k* = 41)	1.3155	0.9730	−0.59 to 3.22	0.1764	
Lifetime substance use				0.1800	0.01%
Less than 5 years (*k* = 44)	Reference				
More than 5 years (*k* = 20)	0.7878	0.5876	−0.36 to 1.92	0.1800	
Assessment period				0.0936	3.54%
Abstinence (*k* = 43)	Reference				
Non‐abstinence (*k* = 72)	−1.3589	0.6285	−2.59 to −0.12	**0.0306**	
Post‐mortem (*k* = 40)	−1.2642	1.4602	−4.12 to 1.59	0.3866	
Age of SUD sample (*k* = 151)	0.0073	0.0553	−0.10 to 0.11	0.8952	0.01%
Age differences between HC and SUD				0.4314	0.26%
Less than 5 years (*k* = 134)	Reference				
More than 5 years (*k* = 17)	1.3074	1.6617	−1.94 to 4.56	0.4314	
Percentage of females in SUD sample (*k* = 152)	0.0190	0.0144	−0.01 to 0.04	0.1875	2.48%
Female % differences between HC and SUD				0.3453	0.85%
Less than 10% (*k* = 115)	Reference				
More than 10% (*k* = 37)	1.3581	1.4390	−1.46 to 4.17	0.3453	

Abbreviations: CI, confidence interval; HC, healthy control; *k*, number of comparisons; SE, standard error; SUD, substance use disorder; VAF, variance accounted for.

Significant *p*‐values are in bold.

To further explore this finding, studies and effect sizes were split based on the percentage of SUD patients who reported regular use of alcohol. Studies in which patients had a drug of preference other than alcohol (such as stimulants or opioids), but in which the percentage of alcohol users was reported, were also included in the analysis. Thus, two groups were created: samples in which all patients reported regular use of alcohol (no. of effect sizes = 61; total *n* for SUD group = 4250) and samples in which not all patients reported regular use of alcohol (no. of effect sizes = 116; total *n* for SUD group = 6280). We found that this dichotomous variable was indeed a significant moderator of heterogeneity, because when all patients reported use of alcohol, higher estimates for oxidant levels were observed. This analysis accounted for almost 9% of the total heterogeneity between oxidant levels and effect sizes.

#### 3.4.2 Biological sample

We found that biological samples were not a significant moderator of heterogeneity in the grouped meta‐analysis of oxidant and antioxidant stress markers. However, for oxidant markers, there was a significant difference between serum and plasma analyses, which suggests that serum was associated with increased effect sizes and larger SMDs when compared to studies that analysed plasma samples.

#### 3.4.3 Lifetime substance use and period of assessment

We found that lifetime substance use and period of assessment were not significant moderators of heterogeneity in the grouped meta‐analysis of oxidant and antioxidant stress markers. However, for antioxidant markers, there was a significant difference between analyses performed with abstinent/non‐abstinent patients. Therefore, abstinence was associated with a decrease in effect sizes when compared with non‐abstinence, suggesting that the effects of SUD on antioxidant defences might be attenuated with drug abstinence.

#### 3.4.4 Age and sex differences

No significant effect of age and percentage of females in SUD samples were found in the grouped meta‐analysis. In addition, no significant effects regarding age differences between controls and SUD samples, as well as regarding female percentage differences between controls and SUD samples, were found.

## 4 DISCUSSION

**FIGURE 3:**
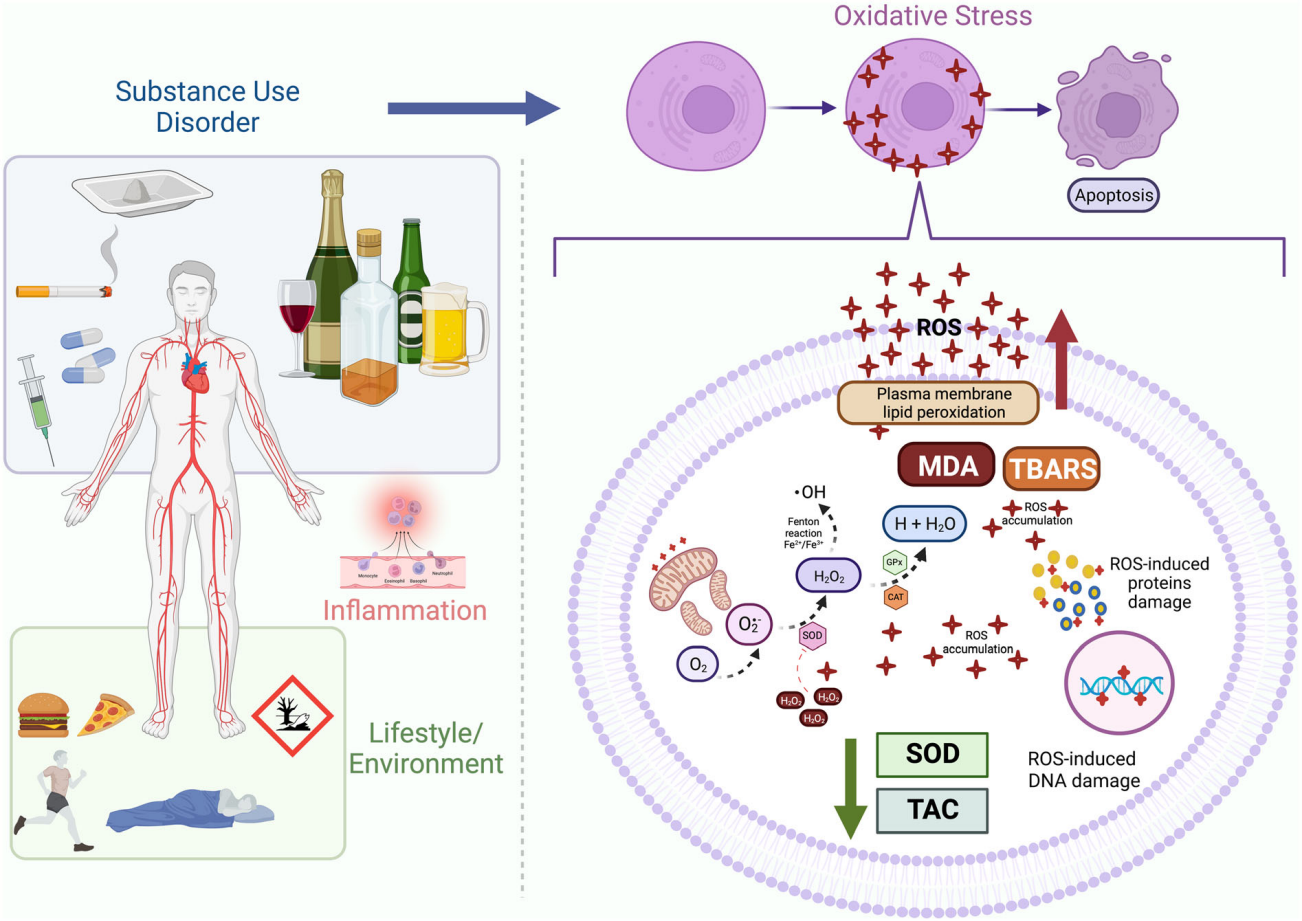
Oxidative stress and substance use disorders. The lifestyle and adverse environmental exposure related to substance use disorders (SUD) are characterized by chronic stress, use of multiple drugs, increased exposure to infections, financial problems, sleep disturbances, insufficient exercise and poor nutrition. All those factors are associated with the activation of the immune system and oxidative stress. NADPH oxidases, xanthine oxidase and the mitochondrial electron‐transport chain can act as active sources of reactive oxygen species (ROS), mainly producing the radical superoxide. SUD can increase reactive oxygen species (ROS) production and negatively affect enzymatic and non‐enzymatic antioxidant capacity. As an effect of this imbalance, biomolecules such as lipids, proteins and DNA can be modified by ROS, having their function disrupted and even ending up in cell death. Participants with SUD, particularly alcohol use disorder, presented elevated levels of lipid damage biomarkers (malondialdehyde [MDA], thiobarbituric acid reactive substances [TBARSs] and lipid peroxidation), reduced superoxide dismutase (SOD) activity and a reduction of total antioxidant capacity (TAC). These results can help to explain the cellular mechanisms and metabolic changes associated with SUD, particularly outcomes related with accelerated ageing. CAT, catalase; Fe, iron; GPx, glutathione peroxidase; H, hydrogen; H_2_O, water; H_2_O_2_, hydrogen peroxide; NADPH, reduced nicotinamide adenine dinucleotide phosphate; O_2_, oxygen; $O_{2}^{•-}$, radical superoxide; ^•^OH, radical hydroxyl; ROS, reactive oxygen species. Created with BioRender.com

In this study, we aimed to assess the effects of SUD on oxidant and antioxidant biomarkers considering clinical and methodological differences. We found higher levels of oxidant makers and lower levels of antioxidants within SUD samples compared with healthy controls on aggregated measures. We also performed individual analyses for 13 distinct biomarkers, with significant results for MDA, TBARS, LP, SOD and TACS. Moreover, estimates were partially moderated by the ‘drug of preference’, ‘biological sample’ and ‘assessment period’ variables. Regarding the ‘drug of preference’ variable, we found a modest difference in oxidant estimates between alcohol and stimulants, with the alcohol showing higher effects. Differences between plasma and serum analyses were also found for oxidant estimates. Finally, abstinence periods were found to impact antioxidants, suggesting more potent effects of SUD on individuals assessed during non‐abstinence than on individuals assessed during abstinence (Figure 3).

Our findings support the idea that chronic licit and illicit substance misuse is associated with OS. These results can help to explain the cellular mechanisms and metabolic changes associated with SUD, particularly outcomes related to accelerated ageing.^7^ Despite the exact reasons still unknown, it is believed that lifestyle and adverse environmental exposure related to SUD could have a major role. They are characterized by chronic stress, use of multiple drugs, increased exposure to infections, financial problems, sleep disturbances, insufficient exercise and poor nutrition.^7^ Whereas the levels of oxidant molecules were shown to increase, the antioxidant system markers were shown to decrease, with potentially insufficient activity to attenuate or prevent the damage caused by free radicals. Hydroxyl (HO˙) and hydroperoxyl (HO_2_) are the main ROS with significant impact on lipid damage. They are produced in high amounts each second and, if not efficiently neutralized, these compounds will quickly attack the biomolecules nearby.^17^, ^18^

We identified MDA, TBARS and LP as the oxidative damage markers that were significantly higher in persons with SUD. They represent three different methods of expressing the outcome of LP, which can be assessed based on the key products generated by the reaction caused by ROS/RNS in the cell membranes. Considering the analysed substances, our review indicated a more significant effect of alcohol consumption on oxidation‐related analytes. This can be partially explained by alcohol metabolization in the liver, which results in hydroxyl radicals, hydrogen peroxide and superoxide. Moreover, there is a subsequent release of toxic compounds into the peripheral circulation,^19^ so these ROS will boost LP.^20^ MDA is the secondary bioproduct continually generated by this process, and it is widely used as a membrane injury indicator.^21^

Additionally, MDA can modify important molecules such as lipids, proteins and DNA, eliciting the proinflammatory process associated with cellular ageing and metabolic disease aetiology, such as obesity and diabetes.^22^, ^23^, ^24^ Another approach to assess the potential damage caused by ROS/RNS is through TBARS. Although TBARSs do not represent a specific and stable biomarker, they have been widely associated with OS damage.^25^ Furthermore, it has already been shown that moderate and high ROS concentrations can contribute to the onset of autophagy, a process closely related to the onset of neurodegenerative diseases.^26^ In that sense, evidence shows that alcohol induces autophagy through ROS production by selectively removing hepatic lipid droplets and defective mitochondria.^27^ Although autophagy is a protective mechanism against ethanol‐induced toxicity, long‐term alcohol exposure might damage this defensive system in many cells, including neurons.^28^

Reinforcing the evidence that SUD can elicit OS, we observed lower SOD activity in persons with SUD. SODs are metalloenzymes located in the cytosol, extracellular space and mitochondria.^29^ They play an essential role in the antioxidant enzymatic system by initiating redox metabolism through the catalyzation of anion superoxide (˙O2−) into hydrogen peroxide (H_2_O_2_) and molecular oxygen O_2_.^30^ The resulting hydrogen peroxide is then reduced to water by other enzymes such as CAT, GSH‐Px and/or the thioredoxin system.^18^, ^31^ SOD is also associated with RNS clearance by encouraging peroxynitrite detoxification (ONOO^−^) and preventing its detrimental effects.^32^ As a result, impairments in SOD activity can culminate in ROS accumulation and cell damage. Some cardiovascular diseases (such as atherosclerosis, hypertension and heart failure) and metabolic diseases are also related to low SOD activity.^33^ In that sense, it is well established that the misuse of substances such as nicotine, alcohol, cocaine, cannabis and other stimulants is associated with increased cardiovascular injury,^34^, ^35^, ^36^, ^37^ which has clinical implications including increased morbidity and mortality associated with SUD.^38^, ^39^

Our meta‐analysis also revealed lower TACS in the SUD group. Non‐enzymatic agents not only participate in enzymatic activity but also integrate the antioxidant system. The first antioxidant protection mechanism relies on the action of bilirubin, ferritin, transferrin, albumin and ceruloplasmin in blood plasma. Dietary antioxidants such as vitamins C and E, carotenoids, reduced GSH and alpha‐lipoic acid are also main components of the non‐enzymatic antioxidant system.^40^ The TACS represents the total amount of antioxidants in biological fluids. Quantifying the TACS can be a useful tool to obtain information on redox status and inform a possible antioxidant supplementation.^41^

The meta‐regression findings should be interpreted cautiously due to the lack of a significant effect in overall moderator analyses, as only pairwise comparisons reached statistical significance. Despite that, it is interesting to note that patients who reported more significant misuse of alcohol also presented higher oxidative damage markers. Alcohol can be metabolized through three pathways: alcohol dehydrogenase, CAT and the microsomal ethanol oxidation system. Each of these pathways can form free radicals. Because ethanol metabolism per se is directly implicated in ROS production, this finding has clinical implications for the treatment of SUDs. Interrupting the intake of illicit drugs is often the focus of detoxification treatments for SUD, whereas alcohol and nicotine take on a secondary role when considering the potential for harm. However, our findings suggest that managing alcohol misuse should be a priority when treating SUD patients, especially those with health comorbidities associated with OS.

Another clinically relevant result refers to the antioxidant system, as higher SUD effects were found among individuals assessed during non‐abstinence periods compared with abstinence phases. Moreover, lower levels of SOD and TACS were observed in persons with SUD. In accordance with our data, other authors^42^ observed that SOD, CAT and GSH‐Px activity was reduced after 14 days of alcohol abstinence. However, others^43^ reported lower SOD activity, but higher CAT and GSH levels in recent abstinent meth users, which reinforces that significant abstinence periods and detoxification treatments are critical for rebalance antioxidant defences.

This study has some limitations that should be discussed. Firstly, we only include four studies that investigated cannabis, one study that investigated inhalants and five studies that investigated polysubstance use without a preferred substance, and these were grouped under the ‘other substances’ category. In addition, most studies that indicated a preferred substance among SUD patients did not provide data regarding polysubstance use, preventing us from carrying out a complete assessment of polydrug use. This is important particularly when it comes to the combined effect of alcohol and stimulants on OS, given that previous evidence already showed that ethanol enhances cocaine's effects on oxidative damage, energetic dysregulation and apoptosis.^44^ In addition, due to a lack of statistical power, we were not able to individually analyse and compare the effects of cannabis, inhalants and poly use in meta‐regression models. However, by grouping them, we collectively compared them with substances that were more often investigated (stimulants, alcohol, nicotine and opioids).

However, previous meta‐analyses have shown that patients with bipolar disorder present significant alterations in antioxidant and oxidant markers, with prominent increases in TBARS and NO levels.^21^ Schizophrenia pathophysiology also has a high interplay with oxidative alterations.^45^, ^46^ More specifically, it has been shown that patients with schizophrenia present increased levels of ROS and reduced SOD and GSH‐Px activity.^47^ Furthermore, subjects with substantial medical comorbidities mixed in the SUD sample were identified in some studies during the screening process. To avoid possible interaction between such diseases and SUD, those studies were not included in the analysis.

The third limitation is the high heterogeneity between studies in our primary analyses. Heterogeneity probably reflects the different methodologies of the analysed studies in terms of eligibility criteria, sampling methods, diagnostic and rating scales, recruitment setting and particularities regarding the measurement of oxidant and antioxidant markers. For instance, our meta‐regression models pointed to differences between plasma and serum analyses when considering estimates of oxidant markers. In addition, given that the publication year of included studies ranged from 1992 to 2020, distinct criteria for clinical diagnosis of SUD were used, including both DSM‐IV and DSM‐V criteria. Finally, there is an underrepresentation of female subjects in our research. Unfortunately, there is still a discrepancy between the number of males and females analysed, so future studies must include both sexes when investigating SUD.

Despite these limitations, it is possible to conclude that persons with SUD show higher oxidative damage markers and lower antioxidant components and that this imbalance is harmful to cells, leading to a wide range of pathologies. It is important to consider the different methodologies used in measuring oxidative damage and antioxidant levels. All these components are quickly degraded or transformed in the biosystem, which underscores the importance of reliable and sensitive methodologies to evaluate OS biomarkers and obtain consistent data. However, we have shown specific markers that are more affected by SUD. Identifying a panel of OS/antioxidant markers specific to SUD could represent another step towards the inclusion of biomarkers as diagnostic and therapeutic tools in the future.

## CONFLICT OF INTEREST

All authors declare no conflicts of interest.

## AUTHOR CONTRIBUTIONS

Thiago Wendt Viola, Rodrigo Orso and Rodrigo Grassi‐Oliveira conceived the study idea. Luisa Fossati Florian, Miguel Gomes Garcia, Marco Giovanni Signor Gomes and Eduarda Mascarenhas Mardini screened the titles, abstracts and texts. Thiago Wendt Viola and João Paulo Ottolia Niederauer performed data analysis. Thiago Wendt Viola, Rodrigo Orso, Aline Zaparte and Rodrigo Grassi‐Oliveira wrote the manuscript. Thiago Wendt Viola, Rodrigo Orso and Rodrigo Grassi‐Oliveira critically revised the manuscript.

## Supporting information


**Table S1.** Individual studies characteristics.

**Figure S1.** Funnel plots ‐ Grouped meta‐analysis of SUD effects on oxidant and antioxidant markers.

## Data Availability

The data that support the findings of this study are available from the corresponding author upon reasonable request.
