# Supplementary material for: Effects of substance use disorder on oxidative and antioxidative stress markers: A systematic review and meta‐analysis
Source: Addict Biol. 2022 Nov 23;28(1):e13254. doi: 10.1111/adb.13254 (PMC10078266; doi:10.1111/adb.13254)
Supplement: Supplementary file 1 — Table S1. Individual studies characteristics. [file ADB-28-0-s002.docx]

**SUPPLEMENTAL MATERIAL**

**List of included studies**

1. Ande A, McArthur C, Ayuk L, et al. Effect of mild-to-moderate smoking on viral load, cytokines, oxidative stress, and cytochrome P450 enzymes in HIV-infected individuals. *PLoS One.* 2015;10(4):e0122402.

2. Al-Akwa AA, Shaher M, Al-Akwa S, Aleryani SL. Free radicals are present in human serum of Catha edulis Forsk (Khat) abusers. *J Ethnopharmacol.* 2009;125(3):471-473.

3. Bani-Ahmad MA, Mustafa AG, Ahmad A, Rahim A. Assessment of oxidative stress of platelets among chronic heroin and hashish addicts. *Hum Exp Toxicol.* 2018;37(10):1017-1024.

4. Bayazit H, Dulgeroglu D, Selek S. Brain-Derived Neurotrophic Factor and Oxidative Stress in Cannabis Dependence. *Neuropsychobiology.* 2020;79(3):186-190.

5. Bayazit H, Selek S, Karababa IF, Cicek E, Aksoy N. Evaluation of Oxidant/Antioxidant Status and Cytokine Levels in Patients with Cannabis Use Disorder. *Clin Psychopharmacol Neurosci.* 2017;15(3):237-242.

6. Dündaröz MR, Türkbay T, Sarici SU, Akay C, Sayal A, Denli M. Selenium and zinc levels in volatile substance abusers. *Biol Trace Elem Res.* 2002;88(2):119-123.

7. Fitzmaurice PS, Tong J, Yazdanpanah M, Liu PP, Kalasinsky KS, Kish SJ. Levels of 4-hydroxynonenal and malondialdehyde are increased in brain of human chronic users of methamphetamine. *J Pharmacol Exp Ther.* 2006;319(2):703-709.

8. Georgakouli K, Manthou E, Fatouros IG, et al. Effects of acute exercise on liver function and blood redox status in heavy drinkers. *Exp Ther Med.* 2015;10(6):2015-2022.

9. Ghazavi A, Mosayebi G, Solhi H, Rafiei M, Moazzeni SM. Serum markers of inflammation and oxidative stress in chronic opium (Taryak) smokers. *Immunol Lett.* 2013;153(1-2):22-26.

10. Govitrapong P, Boontem P, Kooncumchoo P, et al. Increased blood oxidative stress in amphetamine users. *Addict Biol.* 2010;15(1):100-102.

11. Guemouri L, Lecomte E, Herbeth B, et al. Blood activities of antioxidant enzymes in alcoholics before and after withdrawal. *J Stud Alcohol.* 1993;54(5):626-629.

12. Guleken Z, Kuruca SE, Ünübol B, et al. Biochemical assay and spectroscopic analysis of oxidative/antioxidative parameters in the blood and serum of substance use disorders patients. A methodological comparison study. *Spectrochim Acta A Mol Biomol Spectrosc.* 2020;240:118625.

13. Guler EM, Bektay MY, Akyildiz AG, Sisman BH, Izzettin FV, Kocyigit A. Investigation of DNA damage, oxidative stress, and inflammation in synthetic cannabinoid users. *Hum Exp Toxicol.* 2020;39(11):1454-1462.

14. Gutowicz M, Sadurska B, Chołojczyk M, Pokorska-Lis M, Siwińska-Ziółkowska A, Barańczyk-Kuźma A. Antioxidant status in different regions of heroin addicts' brain. *Environ Toxicol Pharmacol.* 2006;21(1):80-85.

15. Hacimusalar, Yunus, Karaaslan O, et al. Methamphetamine’s effects on oxidative stress  markers may continue after detoxification: A case-control study. *Psychiatry and Clinical Psychopharmacology.* 2019;29(3).

16. Huang MC, Chen CC, Peng FC, Tang SH, Chen CH. The correlation between early alcohol withdrawal severity and oxidative stress in patients with alcohol dependence. *Prog Neuropsychopharmacol Biol Psychiatry.* 2009;33(1):66-69.

17. Huang MC, Lin SK, Chen CH, Pan CH, Lee CH, Liu HC. Oxidative stress status in recently abstinent methamphetamine abusers. *Psychiatry Clin Neurosci.* 2013;67(2):92-100.

18. Ikonomidis I, Marinou M, Vlastos D, et al. Effects of varenicline and nicotine replacement therapy on arterial elasticity, endothelial glycocalyx and oxidative stress during a 3-month smoking cessation program. *Atherosclerosis.* 2017;262:123-130.

19. Karademirci M, Kutlu R, Kilinc I. Relationship between smoking and total antioxidant status, total oxidant status, oxidative stress index, vit C, vit E. *Clin Respir J.* 2018;12(6):2006-2012.

20. Kaufman MJ, Streeter CC, Barros TL, et al. Reduced Plasma Nitric Oxide End Products in Cocaine-dependent Men. *J Addict Med.* 2007;1(2):96-103.

21. Kim NH, Kim HC, Lee JY, Lee JM, Suh I. Active and Passive Smoking and Serum Total Bilirubin in a Rural Korean Population. *Nicotine Tob Res.* 2016;18(5):572-579.

22. Kovatsi L, Njau S, Nikolaou K, Tsolakidou A, Karamouzis I, Thisiadou K. Isoprostane as a marker of oxidative stress in chronic heroin users: correlation with duration of heroin use or concomitant hepatitis C infection. *Am J Drug Alcohol Abuse.* 2010;36(1):13-17.

23. Kulkarni S, Ravindra P. Hypomagnesemia in alcohol dependent population from the lower socio-economic background who were consuming illicit liquor and attending the deaddiction center. *World Journal of Pharmaceutical Research.* 2015;4(6).

24. Kulkarni S, Ravindra P, Dhume C, Rodrigues E, Amonkar S. Effects of rehabilitation on biochemical indicators and markers of the  antioxidant system in alcoholic subjects attending de-addiction centre *International Journal of Pharma and Bio Sciences.* 2012;3(1).

25. Kulkarni S, Ravindra P, Dhume C, Rataboli P, Rodrigues E. Levels of plasma testosterone, antioxidants and oxidative stress in  alcoholic patients attending de-addiction centre *Biology and Medicine.* 2009;1(4).

26. Pianca TG, Rosa RL, Ceresér KMM, et al. Differences in biomarkers of crack-cocaine adolescent users before/after abstinence. *Drug Alcohol Depend.* 2017;177:207-213.

27. Sadat-Shirazi MS, Zarrindast MR, Ashabi G. Oxidative stress enzymes are changed in opioid abusers and multidrug abusers. *J Clin Neurosci.* 2020;72:365-369.

28. Salarian A, Kadkhodaee M, Zahmatkesh M, et al. Opioid Use Disorder Induces Oxidative Stress and Inflammation: The Attenuating Effect of Methadone Maintenance Treatment. *Iran J Psychiatry.* 2018;13(1):46-54.

29. Zhou JF, Chen P, Zhou YH, Zhang L, Chen HH. 3,4-Methylenedioxymethamphetamine (MDMA) abuse may cause oxidative stress and potential free radical damage. *Free Radic Res.* 2003;37(5):491-497.

30. Zhou JF, Zhou YH, Zhang L, Chen HH, Cai D. 3,4-methylenedioxymethamphetamine (MDMA) abuse markedly inhibits acetylcholinesterase activity and induces severe oxidative damage and liperoxidative damage. *Biomed Environ Sci.* 2003;16(1):53-61.

31. Al Zyadi M. The Association between Dopamine and Oxidative Stress for  Methamphetamine Iraqi Addicts *Indian Journal of Forensic Medicine & Toxicology.* 2019;13(4).

32. Liu Y, Li H, Wang J, et al. Association of Cigarette Smoking With Cerebrospinal Fluid Biomarkers of Neurodegeneration, Neuroinflammation, and Oxidation. *JAMA Netw Open.* 2020;3(10):e2018777.

33. Mannan SJ, Azad MA, Ullah A, et al. Investigation of serum trace element, malondialdehyde and immune status in drug abuser patients undergoing detoxification. *Biol Trace Elem Res.* 2011;140(3):272-283.

34. Mannelli P, Patkar A, Rozen S, Matson W, Krishnan R, Kaddurah-Daouk R. Opioid use affects antioxidant activity and purine metabolism: preliminary results. *Hum Psychopharmacol.* 2009;24(8):666-675.

35. Mehta AJ, Yeligar SM, Elon L, Brown LA, Guidot DM. Alcoholism causes alveolar macrophage zinc deficiency and immune dysfunction. *Am J Respir Crit Care Med.* 2013;188(6):716-723.

36. Mirecki A, Fitzmaurice P, Ang L, et al. Brain antioxidant systems in human methamphetamine users. *J Neurochem.* 2004;89(6):1396-1408.

37. Molavi N, Ghaderi A, Banafshe HR. Examining Metabolic Profiles in  Opioid-Dependent Patient *International  Journal of Medical Toxicology and Forensic Medicine.* 2020;10(3).

38. Najafi K, Ahmadi S, Rahpeyma M, et al. Study of Serum Malondialdehyde Level in Opioid and Methamphetamine Dependent Patients. *Acta Med Iran.* 2017;55(10):616-620.

39. Narvaez JC, Magalhaes PV, Fries GR, et al. Peripheral toxicity in crack cocaine use disorders. *Neurosci Lett.* 2013;544:80-84.

40. Obianyo O, Liang Y, Burnham EL, et al. Metabolic Consequences of Chronic Alcohol Abuse in Non-Smokers: A Pilot Study. *PLoS One.* 2015;10(6):e0129570.

41. Kotan VO, Yilmaz FM, Neselioglu S, et al. Thiol/Disulphide  Homeostasis in Men with Heroin Addiction *The Journal of Psychiatry and Neurological Sciences.* 2017;30.

42. Paakki P, Stockmann H, Kantola M, et al. Maternal drug abuse and human term placental xenobiotic and steroid metabolizing enzymes in vitro. *Environ Health Perspect.* 2000;108(2):141-145.

43. Panee J, Pang X, Munsaka S, Berry MJ, Chang L. Independent and co-morbid HIV infection and Meth use disorders on oxidative stress markers in the cerebrospinal fluid and depressive symptoms. *J Neuroimmune Pharmacol.* 2015;10(1):111-121.

44. Parthasarathy R, Kattimani S, Sridhar MG. Oxidative stress during alcohol withdrawal and its relationship with withdrawal severity. *Indian J Psychol Med.* 2015;37(2):175-180.

45. Pereska Z, Dejanova B, Bozinovska C, Petkovska L. Prooxidative/antioxidative homeostasis in heroin addiction and detoxification. *Bratisl Lek Listy.* 2007;108(9):393-398.

46. Sandhya C, Sreekumari R, Vidhukumar K. Higher oxidative stress in current alcoholics compared to abstinent alcoholics  and normal controls: a cross-sectional study. *Journal of Evolution of Medical and Dental Sciences.* 2016;5(37).

47. Sapkota M, Burnham EL, DeVasure JM, et al. Malondialdehyde-Acetaldehyde (MAA) Protein Adducts Are Found Exclusively in the Lungs of Smokers with Alcohol Use Disorders and Are Associated with Systemic Anti-MAA Antibodies. *Alcohol Clin Exp Res.* 2017;41(12):2093-2099.

48. Shadnia S, Gorgzadeh N, Soltaninejad K, Abdollahi M, Motevalian S. Status of Total Antioxidant Capacity and Malondialdehyde Level in  Methamphetamine Addicts: A Cross Sectional Study. *International Journal of Medical Toxicology and Forensic Medicine.* 2017;7(1).

49. Shojaeepour S, Fazeli M, Oghabian Z, Pourgholi L, Mandegary A. Oxidative stress in opium users after using lead-adulterated opium: The role of genetic polymorphism. *Food Chem Toxicol.* 2018;120:571-577.

50. Shrestha R, Nepal AK, Lal Das BK, Gelal B, Lamsal M. Non-enzymatic antioxidant status and biochemical parameters in the consumers of Pan Masala containing tobacco. *Asian Pac J Cancer Prev.* 2012;13(9):4353-4356.

51. Solhi H, Malekirad A, Kazemifar AM, Sharifi F. Oxidative stress and lipid peroxidation in prolonged users of methamphetamine. *Drug Metab Lett.* 2014;7(2):79-82.

52. Sordi AO, Pechansky F, Kessler FH, et al. Oxidative stress and BDNF as possible markers for the severity of crack cocaine use in early withdrawal. *Psychopharmacology (Berl).* 2014;231(20):4031-4039.

53. Soykut B, Eken A, Erdem O, et al. Oxidative stress enzyme status and frequency of micronuclei in heroin addicts in Turkey. *Toxicol Mech Methods.* 2013;23(9):684-688.

54. Tretjak Z, Knight J, Wilkins J, Setoda D, Voorhees R. Lipoperoxides in sebum of substance users and controls. *Ann Clin Lab Sci.* 1992;22(4):214-220.

55. Tsai MC, Huang TL. Brain-derived neurotrophic factor (BDNF) and oxidative stress in heroin-dependent male patients undergoing methadone maintenance treatment. *Psychiatry Res.* 2017;249:46-50.

56. Ullah A, Khan A, Iqbal Z, et al. A Clinical Case control study to evaluate Oxidative stress in heroin addicts. *Heroin Addiction and Related Clinical Problems.* 2019;21(6).

57. Vargas HO, Nunes SO, de Castro MR, et al. Oxidative stress and inflammatory markers are associated with depression and nicotine dependence. *Neurosci Lett.* 2013;544:136-140.

58. Woźniak B, Musiałkiewicz D, Woźniak A, et al. Lack of changes in the concentration of thiobarbituric acid-reactive substances (TBARS) and in the activities of erythrocyte antioxidant enzymes in alcohol-dependent patients after detoxification. *Med Sci Monit.* 2008;14(1):CR32-36.

59. Wu SY, Chen CY, Huang TL, Tsai MC. Brain-derived neurotrophic factor and glutathione peroxidase as state biomarkers in alcohol use disorder patients undergoing detoxification. *Medicine (Baltimore).* 2020;99(17):e19938.

60. Zaparte A, Viola TW, Grassi-Oliveira R, da Silva Morrone M, Moreira JC, Bauer ME. Early abstinence of crack-cocaine is effective to attenuate oxidative stress and to improve antioxidant defences. *Psychopharmacology (Berl).* 2015;232(8):1405-1413.

61. Vieira van Keulen H, da Silva Gomes A, Cardoso Fernandes Toffolo M, et al. Serum Concentration Of Nitric Oxide In Women Smokers And Nonsmokers With Overweight. *Nutr Hosp.* 2015;32(4):1493-1499.

**Funnel plots -** *Grouped meta-analysis of SUD effects on oxidant and antioxidant markers*


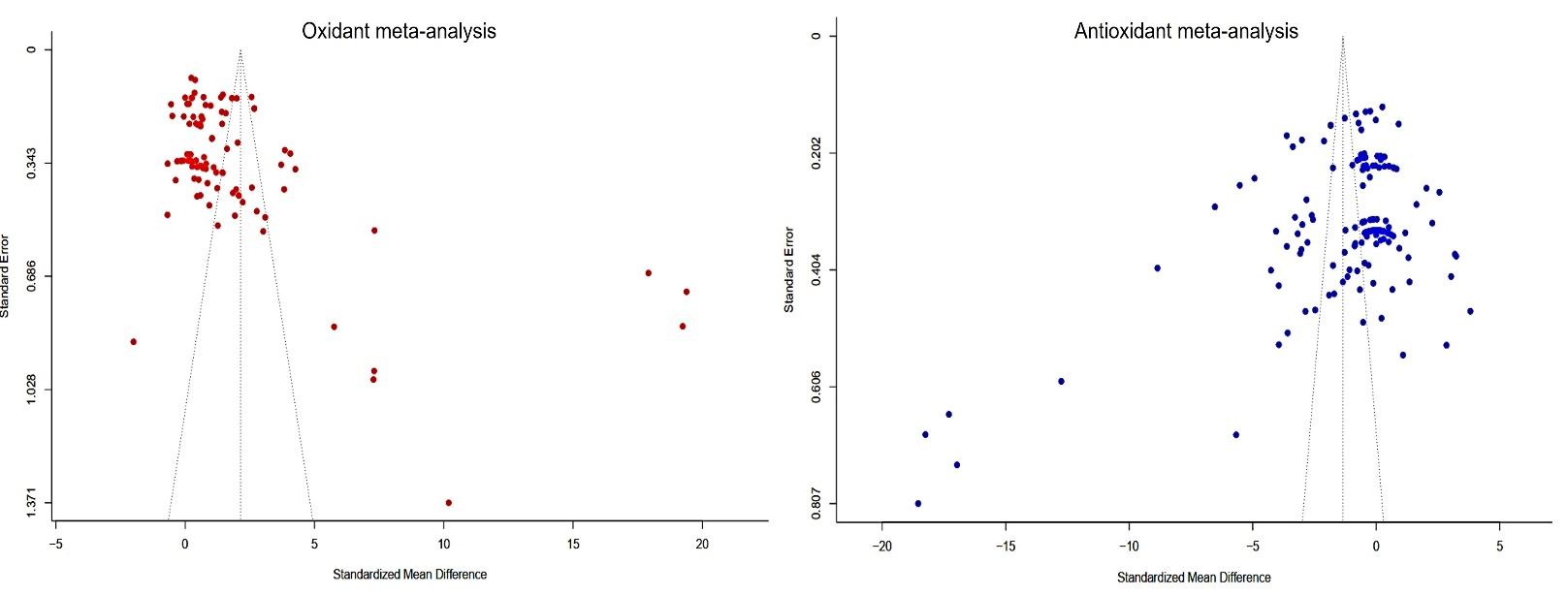


Legend: funnel plots showed that studies were not symmetrically distributed, suggesting that asymmetry could be attributed to publication bias, methodological differences, or true heterogeneity.

| **Individual studies characteristics** | |  |  |  |  |  |
| --- | --- | --- | --- | --- | --- | --- |
| **1° author and year** | **Sample description** | **SUD diagnosis** | **Lab technique** | **Biological sample** | **Assessment time** | **Biomarkers** |
| Obianyo, 2015 | HC n = 10 (80% female; age = 41) SUD n = 10 (20% female; age = 47) | Short Michigan Alcohol Screening Test (Drug of Preference: Alcohol) | HPLC and ELISA | Alveolar macrophages | Abstinence | LP |
| Pannee, 2015 | HC n = 41 (10% female; age = 39) SUD n = 25 (12% female; age = 39) | DSM (Drug of Preference: METH) | ELISA and Spectrophotometry | CSF | Abstinence | GSH and GSH_Px |
| Kovatsi, 2010 | HC n = 22 (0% female; age = 30) SUD n = 42 (0% female; age = 31) | DSM (Drug of Preference: Opioids) | ELISA | Plasma | Abstinence | LP |
| Mannelli, 2009 | HC n = 10 (40% female; age = 35) SUD n = 6 (0% female; age = 36) | DSM (Drug of Preference: Opioids) | HPLC and Biochemical assays | Plasma | Abstinence | GSH |
| Dundaroz, 2003 | HC n = 27 (0% female; age = 15) SUD n = 37 (0% female; age = 15) | DSM (Drug of Preference: Inhalants) | Biochemical assay | Plasma and Erythrocytes | Abstinence | SOD, GSH_Px and MDA |
| Parthasarathy, 2015 | HC n = 50 (0% female; age = 36) SUD n = 50 (0% female; age = 37) | DSM (Drug of Preference: METH) | ELISA | Plasma and Erythrocytes | Abstinence | MDA, PO, CAT and SOD |
| Guemouri, 1993 | HC n = 78 (0% female; age = 35) SUD n = 58 (0% female; age = 42) | DSM (Drug of Preference: Alcohol) | Spectrophotometry | Plasma and Hemolysate | Abstinence | SOD, GSH_Px and CAT |
| Ghazavi, 2013 | HC n = 44 (0% female; age = 31) SUD n = 44 (0% female; age = 30) | DSM (Drug of Preference: Opioids) | ELISA | Serum | Abstinence | TAS/TAC and NO |
| Huang, 2008 | HC n = 19 (0% female; age = 30) SUD n = 76 (0% female; age = 41) | DSM (Drug of Preference: Alcohol) | Spectrophotometry and ELISA | Serum | Abstinence | MDA and SOD |
| Keulen, 2015 | HC n = 18 (100% female; age = 42) SUD n = 20 (100% female; age = 52) | Self-report (Drug of Preference: Nicotine) | Biochemical assay | Serum | Abstinence | NO |
| Kotan, 2017 | HC n = 31 (0% female; age = 26) SUD n = 31 (0% female; age = 26) | DSM (Drug of Preference: Opioids) | Biochemical assays and HPLC | Serum | Abstinence | Thiol |
| Kulkarni, 2015 | HC n = 158 (0% female; age = 38) SUD n = 158 (0% female; age = 37) | CAGE (Drug of Preference: Alcohol) | Spectrophotometry | Serum | Abstinence | GGT |
| Najafi, 2017 | HC n = 22 (-% female; age = -) SUD1 n = 22 (-% female; age = -) SUD2 n = 20 (-% female; age = -) | Self-report (Drug of Preference: 1-Opioids; 2-AMPH) | HPLC | Serum | Abstinence | MDA |
| Wu, 2020 | HC n = 20 (10% female; age = 44) SUD n = 14 (14% female; age = 48) | DSM (Drug of Preference: Alcohol) | ELISA | Serum | Abstinence | SOD, CAT, TBARS, PO and GSH_Px |
| Mehta, 2013 | HC n = 17 (0% female; age = 45) SUD n = 17 (0% female; age = 45) | Short Michigan Alcohol Screening Test (Drug of Preference: Alcohol) | Fluorescence | Alveolar macrophages | Non-abstinent | GSH |
| Liu, 2020 | HC n = 140 (0% female; age = 34) SUD n = 87 (0% female; age = 29) | Self-report (Drug of Preference: Nicotine) | Radioimmunoassay and Spectrophotometry | CSF | Non-abstinent | SOD and NO |
| Soykut, 2013 | HC n = 38 (0% female; age = 33) SUD n = 40 (0% female; age = 34) | DSM (Drug of Preference: Opioids) | Spectrophotometry | Erythrocyte | Non-abstinent | SOD, CAT, GSH_Px and MDA |
| Zhou, 2003a | HC n = 120 (50% female; age = 23) SUD n = 120 (44% female; age = 23) | Urine + Self-report (Drug of Preference: AMPH) | Spectrophotometry | Erythrocytes | Non-abstinent | LP, SOD and CAT |
| Sapkota, 2017 | HC n = 22 (27% female; age = 40) SUD1 n = 22 (31% female; age = 40) SUD2 n = 20 (20% female; age = 43) SUD3 n = 45 (9% female; age = 44) | AUDIT and Self-report (Drug of Preference: 1-Nicotine; 2-Alcohol; 3-Poly) | ELISA | Lung | Non-abstinent | MDA |
| Ande, 2015 | HC n = 11 (50% female; age = 45) SUD n = 11 (5% female; age = 45) | Self-report (Drug of Preference: Nicotine) | ELISA and PCR | Plasma | Non-abstinent | SOD and CAT |
| Bani-Ahmad, 2018 | HC n = 20 (0% female; age = 28) SUD1 n = 20 (0% female; age = 33) SUD1 n = 20 (0% female; age = 24) | Post-mortem (Drug of Preference: 1-Opioids; 2-Cannabis) | Biochemical assay | Plasma | Non-abstinent | PO and TBARS |
| Guler, 2020 | HC n = 40 (0% female; age = 31) SUD n = 40 (0% female; age = 31) | DSM (Drug of Preference: Cannabis) | Spectrophotometry | Plasma | Non-abstinent | TAS/TAC and Thiol |
| Kaufman, 2007 | HC n = 19 (0% female; age = 39) SUD n = 24 (0% female; age = 43) | DSM (Drug of Preference: Cocaine) | Chemiluminescence | Plasma | Non-abstinent | NO |
| Salarian, 2017 | HC n = 20 (0% female; age = 35) SUD1 n = 20 (0% female; age = 36) SUD2 n = 20 (0% female; age = 35) | DSM (Drug of Preference: 1-Opioids; 2-Nicotine) | Spectrophotometry | Plasma | Non-abstinent | SOD, CAT, GSH and MDA |
| Shadnia, 2017 | HC n = 15 (0% female; age = 38) SUD n = 21 (9% female; age = 33) | DSM (Drug of Preference: METH) | Biochemical assays and ELISA | Plasma | Non-abstinent | MDA and TAS/TAC |
| Solhi, 2013 | HC n = 48 (0% female; age = 35) SUD n = 48 (0% female; age = 37) | Post-mortem (Drug of Preference: METH) | Biochemical assay | Plasma | Non-abstinent | MDA and TAS/TAC |
| Vargas, 2013 | HC n = 123 (61% female; age = -) SUD n = 78 (55% female; age = -) | DSM (Drug of Preference: Nicotine) | Biochemical assay | Plasma | Non-abstinent | NO, LP, MDA, PO and TAS/TAC |
| Georgakouli, 2015 | HC n = 17 (-% female; age = 33) SUD n = 17 (-% female; age = 31) | AUDIT (Drug of Preference: Alcohol) | Biochemical assay | Plasma and Erythrocytes | Non-abstinent | GSH, CAT, TAS/TAC and TBARS |
| Govitrapong, 2010 | HC n = 120 (0% female; age = 25) SUD n = 120 (0% female; age = 26) | Urine + Self-report (Drug of Preference: AMPH) | Biochemical assay | Plasma and Erythrocytes | Non-abstinent | TBARS, GSH_Px, CAT and SOD |
| Zhou, 2003b | HC n = 120 (50% female; age = 23) SUD n = 120 (44% female; age = 23) | Urine + Self-report (Drug of Preference: AMPH) | Spectrophotometry | Plasma and Erythrocytes | Non-abstinent | LP, SOD, CAT and GSH_Px |
| Sandhya, 2016 | HC n = 30 (0% female; age = 38) SUD n = 30 (0% female; age = 39) | DSM (Drug of Preference: Alcohol) | Biochemical assay | Plasma and Red blood cells | Non-abstinent | MDA and SOD |
| Ullah, 2019 | HC n = 48 (0% female; age = 36) SUD n = 60 (0% female; age = 33) | Self-Report (Drug of Preference: Opioids) | Spectrophotometry and Biochemical assays | Plasma, Serum and Erythrocytes | Non-abstinent | SOD and CAT |
| Al-Akwa, 2009 | HC n = 20 (0% female; age = 38) SUD n = 20 (0% female; age = 37) | Self-report (Drug of Preference: AMPH) | Biochemical assay | Serum | Non-abstinent | TAS/TAC |
| Bayazit, 2017 | HC n = 34 (0% female; age = 26) SUD n = 34 (0% female; age = 26) | Urine + Self-report (Drug of Preference: Cannabis) | ELISA | Serum | Non-abstinent | TAS/TAC |
| Bayazit, 2019 | HC n = 45 (0% female; age = 25) SUD n = 45 (0% female; age = 25) | Urine + Self-report (Drug of Preference: Cannabis) | ELISA | Serum | Non-abstinent | LP and Thiol |
| Karademirci, 2018 | HC n = 82 (0% female; age = 33) SUD n = 78 (0% female; age = 33) | Fargestrom (Drug of Preference: Nicotine) | Spectrophotometry | Serum | Non-abstinent | TAS/TAC |
| Kulkarni, 2009 | HC n = 160 (0% female; age = 38) SUD n = 200 (0% female; age = 37) | CAGE (Drug of Preference: Alcohol) | ELISA | Serum | Non-abstinent | MDA, PO, SOD, GSH and GR |
| Narvaez, 2013 | HC n = 50 (13% female; age = 33) SUD n = 53 (10% female; age = 30) | DSM (Drug of Preference: Cocaine) | ELISA and Flow Cytometry | Serum | Non-abstinent | TBARS and PO |
| Pakii, 2000 | HC n = 22 (100% female; age = 29) SUD n = 12 (100% female; age = 28) | Urine + Self-report (Drug of Preference: Nicotine) | Biochemical assays and HPLC | Serum | Non-abstinent | GR |
| Shojaeepour, 2018 | HC n = 104 (-% female; age = 47) SUD n = 192 (26% female; age = 50) | Urine + Self-report (Drug of Preference: Opioids) | Spectrophotometry | Serum | Non-abstinent | TBARS, SOD, GSH, TAS/TOC and PO |
| Shrestha, 2012 | HC n = 53 (5% female; age = 31) SUD n = 50 (6% female; age = 33) | Post-mortem (Drug of Preference: Nicotine) | Spectrophotometry | Serum | Non-abstinent | MDA |
| Tretjak, 1992 | HC n = 3 (0% female; age = 40) SUD n = 5 (0% female; age = 36) | Urine + Self-report (Drug of Preference: Poly) | Biochemical assay | Serum | Non-abstinent | MDA |
| Tsai, 2017 | HC n = 30 (0% female; age = 44) SUD n = 60 (0% female; age = 45) | DSM (Drug of Preference: Opioids) | ELISA | Serum | Non-abstinent | SOD, CAT, GSH_Px, TBARS and PO |
| Zyadi, 2019 | HC n = 30 (0% female; age = -) SUD n = 50 (0% female; age = -) | Post-mortem (Drug of Preference: METH) | Biochemical assay | Serum | Non-abstinent | TAS/TAC, NO and LP |
| Molavi, 2020 | HC n = 50 (11% female; age = 39) SUD n = 50 (11% female; age = 39) | Post-mortem (Drug of Preference: Opioids) | ELISA and Spectrophotometry | Serum and Plasma | Non-abstinent | MDA, NO and TAS/TAC |
| Guleken, 2020 | HC n = 15 (0% female; age = 42) SUD n = 30 (0% female; age = 38) | DSM (Drug of Preference: Poly) | Spectrophotometry | Serum and Red blood cells | Non-abstinent | MDA and GSH |
| Ikonomidis, 2017 | HC n = 94 (47% female; age = 49) SUD n = 188 (45% female; age = 50) | Self-report (Drug of Preference: Nicotine) | Spectrophotometry | Plasma | Non-abstinent and Abstinence | MDA and PO |
| Zaparte, 2014 | HC n = 30 (100% female; age = 29) SUD n = 30 (100% female; age = 29) | DSM (Drug of Preference: Cocaine) | Spectrophotometry and Biochemical assays | Plasma | Non-abstinent and Abstinence | PO, SOD, GSH, GSH_Px |
| Wozniak, 2008 | HC n = 20 (0% female; age = 33) SUD n = 42 (0% female; age = 43) | Self-report (Drug of Preference: Alcohol) | Biochemical assay | Plasma and Erythrocytes | Non-abstinent and Abstinence | TBARS, CAT, SOD, GSH_Px |
| Hacimusalar, 2019 | HC n = 45 (0% female; age = 28) SUD n = 44 (0% female; age = 26) | DSM (Drug of Preference: METH) | Biochemical assay | Serum | Non-abstinent and Abstinence | Thiol |
| Huang, 2013 | HC n = 60 (25% female; age = 30) SUD n = 64 (21% female; age = 31) | DSM (Drug of Preference: METH) | Biochemical assay | Serum | Non-abstinent and Abstinence | MDA, SOD, CAT and GSH |
| Kim, 2015 | HC n = 386 (0% female; age = 60) SUD n = 492 (0% female; age = 60) | Self-report (Drug of Preference: Nicotine) | Biochemical assay | Serum | Non-abstinent and Abstinence | GGT |
| Kulkarni, 2012 | HC n = 110 (0% female; age = 37) SUD n = 160 (0% female; age = 36) | CAGE (Drug of Preference: Alcohol) | Spectrophotometry | Serum | Non-abstinent and Abstinence | GGT, SOD, GSH and GSH_Px |
| Mannan, 2010 | HC n = 104 (-% female; age = 30) SUD n = 104 (-% female; age = 30) | DSM (Drug of Preference: Poly) | Spectrophotometry | Serum | Non-abstinent and Abstinence | MDA |
| Pianca, 2017 | HC n = 81 (40% female; age = 15) SUD n = 90 (14% female; age = 16) | DSM (Drug of Preference: Cocaine) | Biochemical assay | Serum | Non-abstinent and Abstinence | TBARS |
| Sordi, 2014 | HC n = 97 (0% female; age = 30) SUD n = 49 (0% female; age = 27) | ASI (Drug of Preference: Cocaine) | Biochemical assay | Serum | Non-abstinent and Abstinence | TBARS |
| Pereska, 2007 | HC n = 22 (22% female; age = 23) SUD n = 17 (17% female; age = 24) | Urine + Self-report (Drug of Preference: Opioids) | Spectrophotometry | Serum and Plasma | Non-abstinent and Abstinence | MDA and TAS/TAC |
| Fitzmaurice, 2006 | HC n = 21 (9% female; age = 33) SUD n = 16 (31% female; age = 32) | Urine + Self-report (Drug of Preference: METH) | Spectrophotometry | Brain | Postmortem | MDA and LP |
| Gutowicz, 2006 | HC n = 8 (12% female; age = 24) SUD n = 9 (11% female; age = 28) | Post-mortem (Drug of Preference: Opioids) | Spectrophotometry | Brain | Postmortem | MDA, PO, SOD and CAT |
| Mirecki, 2004 | HC n = 16 (12% female; age = 35) SUD n = 20 (25% female; age = 31) | Post-mortem (Drug of Preference: METH) | HPLC | Brain | Postmortem | GSH, GSH_Px, SOD, GR and GGT |
| Sadat-Shirazi, 2019 | HC n = 13 (0% female; age = 36) SUD1 n = 56 (0% female; age = 38) SUD2 n = 45 (0% female; age = 35) | Post-mortem (Drug of Preference: 1-Opioids; 2-Poly) | Western blot and Spectrophotometry | Brain | Postmortem | MDA and SOD |

*Legend*: Healthy control (HC); Substance use disorder SUD; Sample size (n); Mean group age (age); Methamphetamine (METH); Amphetamine (AMPH); Cerebrospinal fluid (CSF); Addiction Severity Index (ASI); Diagnostic and Statistical Manual of Mental Disorders (DSM); Thiol levels (Thiol); Superoxide dismutase (SOD); Nitric Oxide (NO); Lipid peroxidation (LP); Total antioxidant capacity/status (TAS/TAC); Malondialdehyde (MDA); Protein oxidation (PO); Thiobarbituric acid reactive substances (TBARS); Catalase (CAT); Reduced glutathione (GSH); Glutathione peroxidase (GSH-Px); Glutathione reductase (GR); Gamma-glutamyl transferase (GGT); Missing data (-).
