# Supplementary figures and images for: Effects of substance use disorder on oxidative and antioxidative stress markers: A systematic review and meta‐analysis
Source: Addict Biol. 2022 Nov 23;28(1):e13254. doi: 10.1111/adb.13254 (PMC10078266; doi:10.1111/adb.13254)

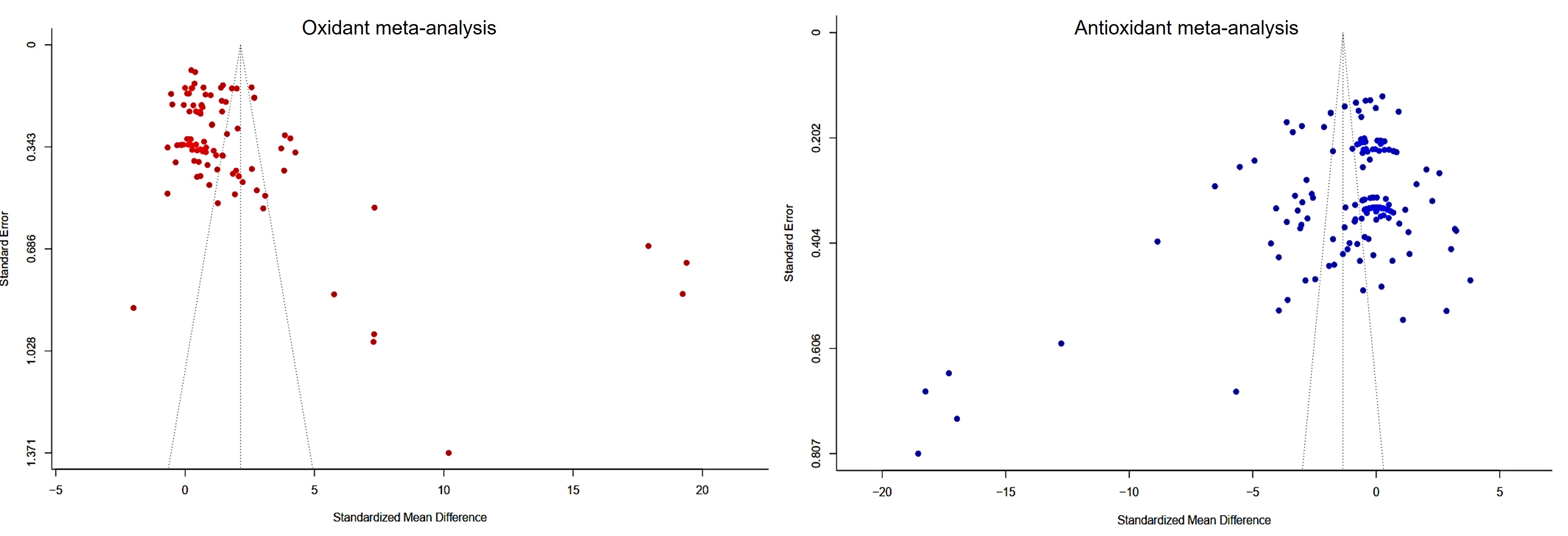

Supplement: Supplementary file 2 — Figure S1. Funnel plots ‐ Grouped meta‐analysis of SUD effects on oxidant and antioxidant markers. [file ADB-28-0-s001.jpg]
